# The effect of restrictive versus liberal selection criteria on survival in ECPR: a retrospective analysis of a multi-regional dataset

**DOI:** 10.1186/s13049-023-01154-1

**Published:** 2023-12-04

**Authors:** Arne Diehl, Andrew C. Read, Timothy Southwood, Hergen Buscher, Mark Dennis, Vinodh Bhagyalakshmi Nanjayya, Aidan J. C. Burrell

**Affiliations:** 1 Department of Intensive Care and Hyperbaric Medicine, The Alfred Hospital, Commercial Road, Melbourne, Australia; 2 Australian and New Zealand Intensive Care Research Centre (ANZIC-RC), Department of Epidemiology and Preventive Medicine, Monash University, Melbourne, Australia; 3 Department of Intensive Care, St Vincent’s Hospital, Sydney, Australia; 4 Department of Intensive Care, Royal Prince Alfred Hospital, Sydney, Australia; 5 Department of Cardiology, Royal Prince Alfred Hospital, Sydney, Australia; 6 Faculty of Medicine and Health, University of Sydney, Sydney, Australia

**Keywords:** Cardiac arrest, In-hospital cardiac arrest, Out-of-hospital cardiac arrest, Extracorporeal membrane oxygenation, ECPR, ECMO

## Abstract

**Background:**

Extracorporeal cardiopulmonary resuscitation (ECPR) is an established rescue therapy for both out-of-hospital cardiac arrest (OHCA) and in-hospital cardiac arrest (IHCA). However, there remains significant heterogeneity in populations and outcomes across different studies. The primary aim of this study was to compare commonly used selection criteria and their effect on survival and utilisation in an Australian ECPR cohort.

**Methods:**

We performed a retrospective, observational study of three established ECPR centres in Australia, including cases from 1 January 2013 to 31 December 2020 to establish the baseline cohort. We applied five commonly used ECPR selection criteria, ranging from restrictive to liberal.

**Results:**

The baseline cohort included 199 ECPR cases: 95 OHCA and 104 IHCA patients. Survival to hospital discharge was 20% for OHCA and 41.4% for IHCA. For OHCA patients, strictly applying the most restrictive criteria would have resulted in the highest survival rate 7/16 (43.8%) compared to the most liberal criteria 16/73 (21.9%). However, only 16/95 (16.8%) in our cohort strictly met the most restrictive criteria versus 73/95 (76.8%) with the most liberal criteria. Similarly, in IHCA, the most restrictive criteria would have resulted in a higher survival rate in eligible patients 10/15 (66.7%) compared to 27/59 (45.8%) with the most liberal criteria. With all criteria a large portion of survivors in IHCA would not have been eligible for ECMO if strictly applying criteria, 33/43 (77%) with restrictive and 16/43 (37%) with the most liberal criteria.

**Conclusions:**

Adherence to different selection criteria impacts both the ECPR survival rate and the total number of survivors. Commonly used selection criteria may be unsuitable to select IHCA ECPR patients.

**Supplementary Information:**

The online version contains supplementary material available at 10.1186/s13049-023-01154-1.

## Background

Extracorporeal cardiopulmonary resuscitation (ECPR) has become an established therapy for refractory cardiac arrest globally [1–3]. Despite the increasing use of ECPR, survival rates vary significantly across different studies. Recent randomised trials have reported survival rates for out-of-hospital cardiac arrest (OHCA) between 20 and 43% [4–6], while large international registries report survival for OHCA treated with ECPR at lower levels between 8.4 and 16.4% [7–9].

One key factor is that there is no international consensus on the ideal selection criteria for ECPR, resulting in significant heterogeneity across populations in different studies. This includes variations in key prognostic variables such as the type of arrest, rhythm, timeline and patient age of the included populations [7, 10, 11]. Similarly, the cardiac arrest characteristics of in-hospital cardiac arrest (IHCA) may differ greatly from out-of-hospital populations [12, 13].

There is also uncertainty how the variation in selection criteria impacts patient outcomes. Conceptually, liberal inclusion criteria result in more patients being included, but lower survival rates for the program, while restrictive criteria would result in less patients being included but higher survival rates. We therefore sought to (1) assess the impact on ECPR survival of commonly used selection criteria on a multi-regional dataset; and (2) compare the utility of different selection criteria for both in- and out-of-hospital cardiac arrest.

## Methods

### Study design and population

This was a multi-centre retrospective observational study analysing the ability of various ECPR selection criteria to differentiate survivors and non-survivors from ECPR. Included were all patients who received ECPR from 1 January 2013 to 31 December 2020 at one of the three participating metropolitan centres in Sydney and Melbourne, Australia. The centres have long established ECPR programs that have existed since the start of the study period. All three sites had similar inclusion criteria derived from the CHEER criteria, however adherence to criteria was not mandated, so that patients outside of these criteria were frequently included. All patients receiving ECMO had data routinely collected in local databases used for data submission to Extracorporeal Life Support Organisation (ELSO) Registry. Patients who had insufficient data on the selection criteria, patients with traumatic cardiac arrest, accidental hypothermia or drowning were excluded. We also excluded patients who had received ECMO more than 20 min after return of spontaneous circulation (ROSC) according to the ELSO definition for ECPR.

### Definitions and criteria

Differentiation between OHCA and IHCA occurred based on the site of the initial cardiac arrest. The start of the arrest was assumed to be the ambulance call time for OHCA or time recorded if the arrest occurred while the ambulance was in attendance. All OHCA patients were cannulated after arrival to the hospital. Time of the cardiac arrest code was assumed to be the time of cardiac arrest for IHCA. Any period of ROSC during the cardiac arrest of less than 20 min was considered continuous cardiac arrest time. Re-arrest after a period of > 20 min of ROSC was considered as a separate cardiac arrest and the longer cardiac arrest time was chosen for the analysis. For the initial rhythm we equated AED delivered shocks as shockable. Witnessed was defined as either auditory or visual. Bystander ‘CPR performed’ was defined as specified with each set of criteria according to the delay from arrest until CPR was initiated in minutes.

Five sets of ECPR selection criteria were applied retrospectively to our dataset and were chosen by consensus by the group of authors from publications relevant to the Australian setting. These included the criteria from three randomised controlled trials (ARREST, Prague, INCEPTION), one observational study (CHEER) and one local policy (Alfred Hospital) [1, 4–6, 14]. The composition of the criteria is shown in the Table 1. Differences in the description of the selection criterion ‘no end-stage disease’ or a similarly named criterion describing the same concept are listed in the Additional file 1: Table A1. Based upon the fulfilment of the applied ECPR selection criteria a status of included (a patient would have been offered ECPR) or excluded (a patient would not have been offered ECPR) was assigned to both survivors and non-survivors in our dataset.

**Table 1:**
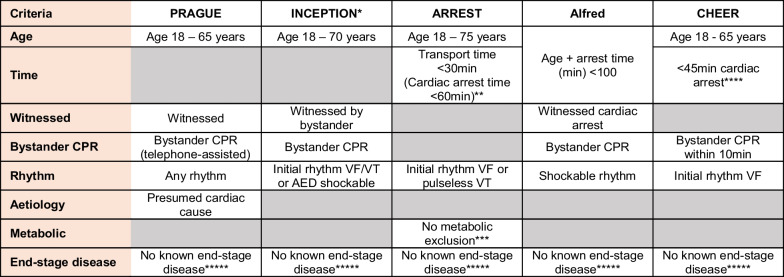
ECPR inclusion criteria selected for analysis

^*^At the time of randomization in the field, patients were excluded if they had an anticipated time from cardiac arrest to start of cannulation of > 60 min. However, once in the emergency department, patients already randomized were not excluded if > 60 min at point of cannulation

^**^No prescribed time limit however < 60 min was based on sum of mean times: call to ambulance arrival (6 min) + EMS scene time (22 min) + inclusion criteria < 30 min transport time, therefore approximately 60 min cut off. Other logistical criteria: able to fit CPR device and exclusion if cath lab unavailable or absolute CI to emergent angiography (contrast allergies; active GI or internal bleeding)

^***^Metabolic exclusion criteria (applied after randomization but ECMO not initiated) two or more of the following: end-tidal CO_2_ < 10 mm Hg, PaO_2_ < 50 mm Hg or oxygen saturation < 85%, and lactate > 18 mmol/L

^****^Physician discretion regarding timing, informal < 45 min to achieve target of ECMO support by 60 min; a mechanical CPR device needed to be available

^*****^Variations of the definition of end-stage disease (or similar wording) are listed in the appendix (Additional file 1: Table A1)

Shaded squares represent not specified characteristics

Only patients with the full basic characteristics of all selection criteria available were included with the exception of the metabolic inclusion criteria cited in the ARREST trial (at least two out of three, end-tidal carbon dioxide (CO_2_) > 10 mmHg, arterial oxygen pressure (PaO_2_) > 50 mmHg or arterial oxygen saturation > 85%, lactate < 18 mmol/L). The metabolic criteria could only be applied if the data was available, whereby the lactate and PaO_2_ was taken from the blood gas closest to ECMO initiation and not necessarily pre-ECMO, and the end-tidal CO_2_ only if recorded at the time. These data points are available in the Additional file 1: Table A2.

We defined liberal as criteria leading to the greatest number of patients eligible for ECPR on our dataset and restrictive criteria with the least number of patients eligible by strict application of the criteria.

### Ethics and consent

The study was approved as low risk project (476/20) under the National Mutual Acceptance scheme by the Ethics Committee of the Alfred Hospital, Melbourne (Australia) and received a waiver of consent.

### Data collection and analysis

De-identified data was collected and submitted to a central REDCap database hosted by Monash University, Melbourne. Online meetings for all data entry personnel were held to ensure consistency in data collection according to the detailed data dictionary. Quality checks of critical data points entered into the data form and clarification for inconsistencies were performed. All data were analysed with STATA 14.2 (StataCorp. College Station, TX, USA: StataCorp LP). Categorical variables were described as proportions with percentage and were compared using chi-square. Continuous variables were described as mean and standard deviation or median with interquartile range based on the distribution of data. Comparisons of continuous variables were performed using one-way ANOVA or Mann–Whitney test, as appropriate. A *p*-value of < 0.05 was considered significant.

## Results

**Fig. 1:**
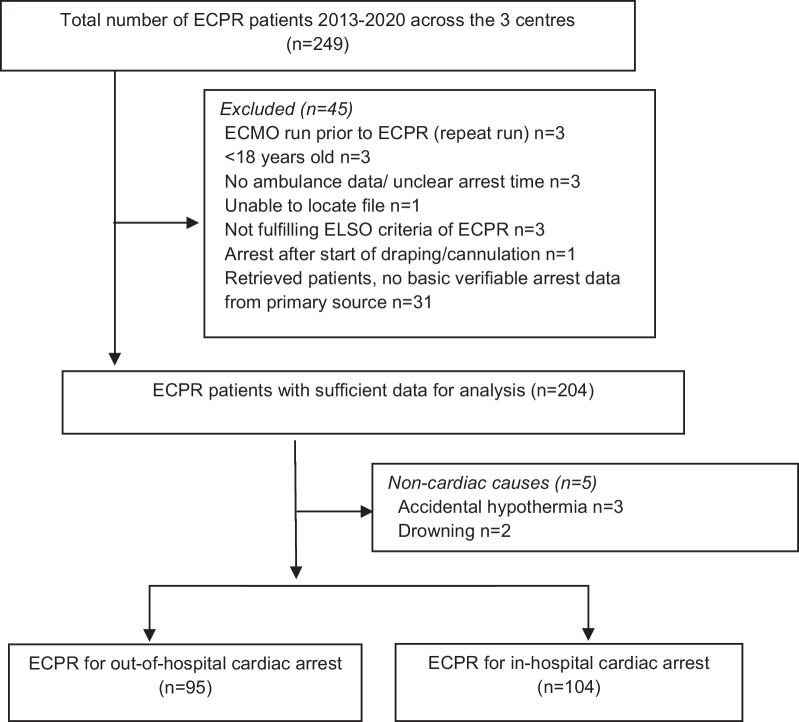
Consort diagram. Total number of ECPR patients, the number and reason for exclusion, non-cardiac arrests and the separation by location of arrest. ECPR: extracorporeal cardiopulmonary resuscitation, ECMO: extracorporeal membrane oxygenation, ELSO: Extracorporeal Life Support Organisation

A total of 249 patients received ECPR from 1 January 2013 to 31 December 2020. 45 patients were removed with incomplete data. Five patients with non-cardiac arrests were excluded leaving a total of 199 patients in the final analysis, 104 (52%) IHCA and 95 (48%) OHCA (Fig. 1). Differences in baseline characteristics, treatments and outcomes between IHCA and OHCA are displayed in Table 2. Inpatients were older with a higher burden of comorbidities, more prior cardiac interventions and a lower rate of initial shockable rhythms (35.7% vs. 72.3%, *p* < 0.001). Survival to hospital discharge was 41.4% for IHCA and 20% for OHCA, with 100% and 89.5% having a favourable neurological outcome (CPC 1 or 2) respectively. Patients with IHCA had a shorter time to ECMO support than with OHCA (median 39 min vs. 77 min, *p* < 0.001) with shorter cardiac arrest time to arrival of the ECMO team (median 10 min vs.50 min, *p* < 0.001). The median ECMO duration to weaning was similar between 5 and 4 days respectively. Organ donation occurred more frequently with OHCA.

**Table 2:** Baseline, cardiac arrest times, ECMO run and outcome characteristics of ECPR patients, in-hospital versus out-of-hospital cardiac arrest

Characteristics	Total (n = 199)	In-hospital (n = 104)	Out of hospital (n = 95)	P-value
Age, yr, mean (SD)	50.5 (15.2)	52.6 (16.0)	48.2 (14.1)	0.02
Female, *n* (%)	48 (24.1)	33 (31.7)	15 (15.8)	0.009
*Comorbidities,* *n* (%)				
Diabetes type 1 or 2	33 (16.6)	23 (22.1)	10 (10.5)	0.028
Chronic kidney disease CKD 1–3	9 (4.5)	9 (8.7)	0 (0)	0.003
Heart failure (NYHA 1 or 2)	8 (4.0)	6 (5.7)	2 (2.1)	0.19
Peripheral vascular disease	5 (2.5)	4 (3.8)	1(1.0)	0.21
Ischaemic heart disease (prior to admission)	29 (14.6)	21 (20.2)	8 (8.4)	0.019
Cardiac intervention prior (PCI, CABG, other)	40 (20.1)	34 (32.7)	6 (6.3)	< 0.001
COPD (Stage 3 or less)	5 (2.5)	4 (3.9)	1 (1.0)	0.21
Chronic liver disease (Child–Pugh A or less)	3 (1.5)	1 (1.0)	2 (2.1)	0.53
*Cardiac arrest and ECMO run*				
CPR in progress at ECMO initiation, *n* (%)	187 (94)	98 (94.2)	89 (93.7)	0.87
Shockable initial rhythm, *n* (%)	103 (53.7)	35 (35.7)	68 (72.3)	< 0.001
Any ROSC, *n* (%)	86 (43.2)	46 (44.2)	40 (42.1)	0.76
Bystander CPR performed, *n* (%)	191 (96.0)	104 (100)	87 (91.6)	0.003
Witnessed arrest, *n* (%)	185 (93.0)	100 (96.2)	85 (89.5)	0.066
Duration of cardiac arrest* at arrival of ECMO provider, min, median, (IQR)	30 (8–51)	10 (5–25)	50 (43–66)	< 0.001
Time for decision, set up, cannulation, min, median, (IQR)	22 (16–35)	21 (13–35)	25 (20–34)	0.022
Total time to ECMO support, min, median, (IQR)	59 (35–82)	39 (24–53)	77 (68–95)	< 0.001
*Outcome*				
Coronary angiogram performed, *n* (%)	120 (60.3)	53 (51.0)	67 (70.5)	0.005
*Cardiac therapeutic intervention, n (%)*				
Percutaneous coronary intervention** review	65 (32.7)	26 (25.0)	39 (41.1)	0.016
Cardiac bypass surgery	13 (6.5)	10 (9.6)	3 (3.1)	0.066
AICD	13 (6.5)	9 (8.67	4 (4.2)	0.21
Other	26 (13.1)	17 (16.4)	9 (9.5)	0.15
ECMO weaned sucessfully, *n* (%)	88 (44.2)	63 (60.6)	25 (26.3)	< 0.001
ECMO duration (weaned), d, median (IQR)	4 (2–7)	5 (2–8)	4 (2–6)	0.54
Bridge to LVAD, *n* (%)	5 (2.5)	4 (3.9)	1 (1.0)	0.21
Bridge to heart transplant, *n* (%)	4 (2.0)	1 (1.0)	3 (3.2)	0.27
Survival to hospital discharge, *n* (%)	62 (31.2)	43 (41.4)	19 (20.0)	0.001
*CPC of survivors at discharge, total n = 62,* *n* (%)				
1	51 (82.2)	38 (88.4)	13 (68.4)	
2	9 (14.5)	5 (11.6)	4 (21.1)	
3	1 (1.6)	0 (0)	1 (5.3)	
4	0 (0)	0 (0)	0 (0)	
Uncertain CPC	1 (1.6)	0 (0)	1 (5.3)	
Organ donation, *n* (%)	14 (7.0)	3 (2.9)	11 (11.6)	0.017

^*^cardiac arrest time refers to the longest arrest time with or without ROSC < 20 min

*SD* Standard deviation, *IQR* Interquartile range, *CKD* Chronic kidney disease, *NYHA* New York Heart Association, *PCI* Percutaneous coronary intervention, *CABG* Coronary artery bypass grafting, *COPD* Chronic obstructive pulmonary disease, *ROSC* Return of spontaneous circulation, *CPR* Cardiopulmonary resuscitation, *ECMO* Extracorporeal membrane oxygenation, *LVAD* Left ventricular assist device, *AICD* Automated internal cardiac defibrillator, *CPC* Cerebral performance category

### Relationship between survival and selection criteria

**Fig. 2:**
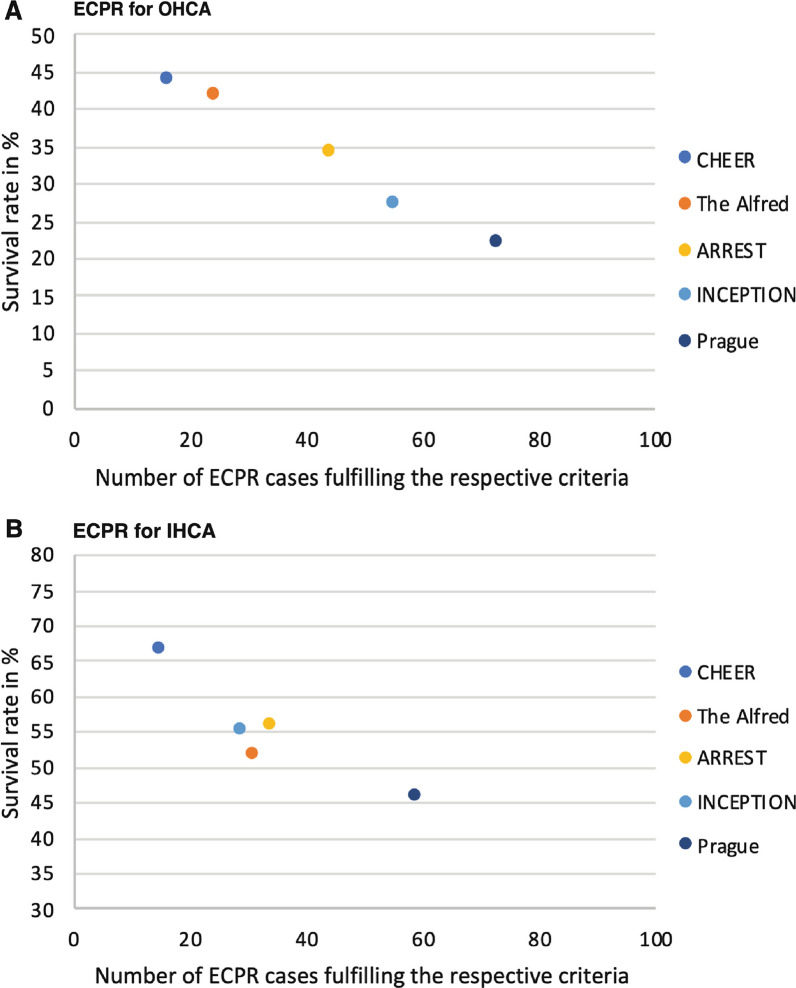
Relationship between survival and number of cases fulfilling all respective criteria. ECPR for OHCA (**A**) and IHCA (**B**) both show an inverse relationship between survival rates and the number of cases performed strictly fulfilling the respective inclusion criteria. ECPR, extracorporeal cardiopulmonary resuscitation; OHCA, out-of-hospital cardiac arrest; IHCA, in-hospital cardiac arrest

The applied selection criteria [1, 4–6, 14] are summarised in Table 1, ranging from liberal criteria to more restrictive criteria (in respective order Prague, INCEPTION, ARREST, The Alfred, CHEER). For OHCA patients (Fig. 2A), retrospectively applying the most restrictive criteria would have resulted in higher survival 7/16 (43.8%) compared to the most liberal criteria 16/73 (21.9%). Inversely, the number of patients fulfilling all criteria and therefore eligible would have included only 16/95 (16.8%) of our cohort with the most restrictive criteria and 73/95 (76.8%) with the most liberal criteria. Similarly, in IHCA (Fig. 2B), more restrictive criteria resulted in a higher survival rate 5/15 (66.7%) versus 27/59 (45.8%), but a lower number of eligible patients fulfilling all criteria 15/105 (14.3%) versus 59/105 (56.2%), as compared to more liberal criteria. An inverse relationship between the survival rate and the number of eligible cases was present for both OHCA and IHCA (Fig. 2).

### Distribution of survivors and non-survivors

**Fig. 3:**
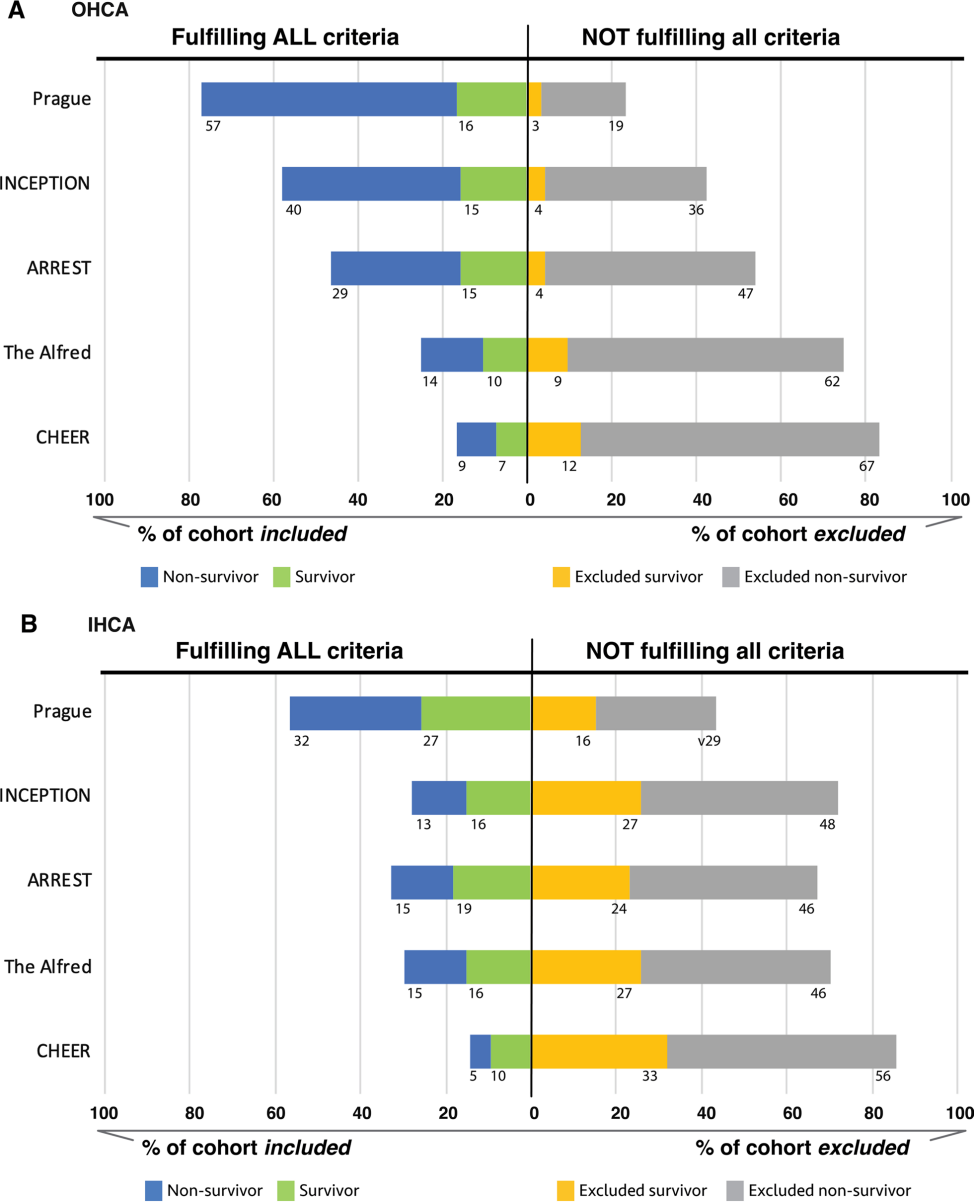
Distribution of survivors and non-survivors of ECPR for OHCA (**A**) and IHCA (**B**). Cohort analysed for different ECPR selection criteria (Table 1). Displayed are the percentages of this cohort fulfilling *ALL* inclusion criteria (survivors in green, non-survivors in blue) and patient *NOT* fulfilling all inclusion criteria (survivors in yellow, non-survivors in grey) who would not have been started on ECPR with strict criteria application. Bars represent percentages, absolute patient numbers are displayed adjacent to the bar. OHCA, out-of-hospital cardiac arrest; IHCA, in-hospital cardiac arrest

The number of patients eligible for ECPR, as well as the number excluded, depended on the applied criteria and if it was for OHCA or IHCA. As can be seen in Fig. 3, for OHCA (A), limiting ECPR to the most restrictive criteria would have resulted in only 7/19 (36.8%) of all survivors meeting criteria (green bar/ green and yellow bars) versus 16/19 (84.2%) with liberal criteria. Among the large number of ineligible patients with the most restrictive criteria 79/95 (83.2%) were the potential missed survivors (yellow bar) 12/19 (63%) of all survivors. On the other hand, using the most liberal criteria would have resulted in a smaller fraction of ineligible cases 22/95 (23.2%). This would have also translated to a lower number of potential missed ECPR survivors (yellow bar), with 3/19 15.8% of all survivors not included.

For IHCA (Fig. 3B), adjusting the different selection criteria had a similar directional impact on the number of eligible survivors, however this effect was more pronounced. Using restrictive criteria, only 16/45 (35.5%) of all survivors were included (green bar/ green and yellow bars), as compared to more liberal criteria 33/45 (73.3%) of all survivors. Conversely among the large number of excluded patients with IHCA, an even larger fraction 16/43 (37%)–33/43 (77%) of all ECPR survivors would have been excluded for ECMO in this cohort applying the respective selection criteria to IHCA patients.

**Fig. 4:**
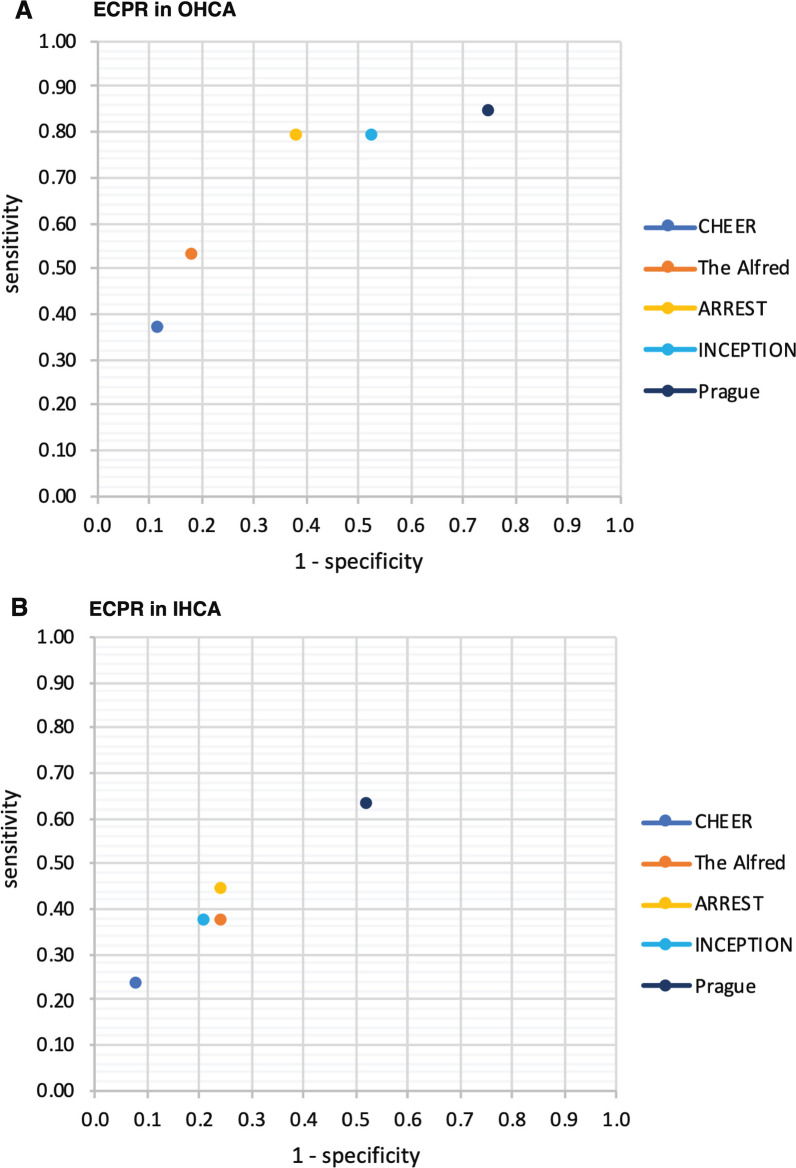
Sensitivity and 1-specificity of selected ECPR criteria for survival to hospital discharge. Characteristics of sensitivity and 1-specificity displayed for OHCA (**A**) and IHCA (**B**) for all retrospectively applied selection criteria to our dataset (criteria are detailed in Table 1). OHCA, out-of-hospital cardiac arrest; IHCA, in-hospital cardiac arrest

The test characteristics for each set of criteria in their ability to identify survivors were plotted onto a sensitivity and specificity grid in Fig. 4. For OHCA (Fig. 4A) the criteria resemble at best moderately performing characteristics and for IHCA (Fig. 4B) approach random differentiation between survivors and non-survivors (details Additional file 1: Table A3).

## Discussion

### Key findings

In this study, we retrospectively applied five alternative ECPR selection criteria to an ECPR dataset from three metropolitan ECPR centres in Australia. We found that applying different selection criteria had a large impact on the population included and survival rates. We showed that more restrictive criteria could potentially result in (1) a reduction of the total number of ECPR cases performed (2) a higher survival rate in those cases that are performed but (3) could potentially also increase the number of missed survivors that did not fulfil all criteria but may have still survived. The reverse is true for more liberal criteria. We also found that a greater proportion of the IHCA ECPR population would have been excluded from ECPR compared to OHCA if strictly adhering to the current selection criteria.

### Relationship to previous OHCA studies

The survival rates in this study, when applying the five various selection criteria, demonstrated similar trends to the original trials they were based upon. The major limitation being that we were not able to review patient level data for comparison instead we compared by selection criteria only. The dataset is consistent but it represents ‘real-life’ data and is not prospective. The PRAGUE trial selection criteria included patients most liberally in our cohort. We found applying these criteria resulted in the greatest number of survivors, but also more non-survivors, with overall the lowest survival rate. For OHCA the survival rates in our analysis were similar to the original study. It showed 39/124 (31.5%) neurologically intact survival (CPC1 or 2) at 180 days, as compared to 16/73 (21.9%) survival to discharge for all patients fulfilling the Prague criteria in this cohort. The most restrictive criteria were the CHEER criteria, with age < 65 limit, arrest time limit to < 45 min and being limited to VF alone. This resulted in a survival rate of 7/16 (43.8%), which was similarly high compared to the original study 3/9 (33.3%).

The INCEPTION criteria included restrictive variables (witnessed by bystander, bystander CPR and an initial shockable rhythm). However, despite early transport and an intention to exclude patients in the field with an anticipated start to cannulation > 60 min, the actual delay to start of cannulation hindered a restrictive time criterion and a large number of patients were cannulated > 60 min post arrest. 14/70 (20%) survived in the intention-to-treat ECPR arm, however only 46 of these patients received ECPR with a survival of 5/46 (10.9%) compared to 15/55 (27.3%) applying the INCEPTION criteria to this dataset. The median time to ECMO support was very similar at 74 min (vs. 77 min in our cohort) suggesting that system factors such as first responders, pre-hospital care and experience in simultaneous advanced life support and ECPR cannulation play a vital role for outcomes.

### The comparison between IHCA and OHCA ECPR

Randomised data demonstrating survival benefit for ECPR in IHCA is lacking. However, survival rates for in-hospital populations are described in the range of 20–40% [15, 16], substantially higher than the comparable ECPR survival form registry data for OHCA 8.4–16.4% [7–9].

Overall, survival following IHCA ECPR was two times that for OHCA in our cohort. By strictly applying the selection criteria, even higher survival post ECPR for IHCA was achieved. Restrictive criteria (like the CHEER criteria) applied to our data resulted in 10/15 (66.7%) survival versus 9/15 (60%) in the original study at the cost of excluding a group of patients from ECMO that may still have benefited from ECPR support.

Despite the fact that in-hospital cardiac arrest patients were on average 4 years older and had more comorbidities and a lower rate of shockable rhythms, the survival outcomes were substantially better than for OHCA (see Table 2). We speculate that the shorter time to establish ECPR, a well described prognostic variable, coupled with immediate trained CPR in the hospital setting, likely accounts for a substantial portion of this difference. Survival predictors for ECPR in the in-hospital setting have been recently described in the (RESCUE-IHCA) score [17]. Aside from in-hospital illness categories (medical cardiac vs surgical cardiac vs surgical non-cardiac) and renal insufficiency all other factors have been previously described: age, duration of cardiac arrest, time of day and presenting rhythm. The only moderate performance of the predictive score raises the question whether entirely different criteria are needed for IHCA, and should be an area of future research.

### Strengths and limitations

This study has several strengths. We included a fairly large, multiregional cohort of ECPR patients, which improves the generalisability of the findings. We developed clear definitions a priori to reduce bias in data collection. Three experienced researchers used multiple primary sources to cross check the validity of the data. However, there are several limitations. The decision to initiate ECMO is complex, and additional unmeasured factors may have influenced clinicians. The prehospital and ECPR practise itself was not mandated in this observational study—however practice is similar across both states of Australia. Finally, many patients were initiated on ECPR despite being outside local protocols. This can be seen in that > 20% of patients did not fulfil even the liberal criteria. This unpredictable application of the criteria may have influenced the final cohort and the overall findings of the study. Future work will focus on the impact of different criteria on ECPR usage and outcomes.

### Implications

For ECPR criteria to be effective in including most potential survivors, the data need to be both simple and readily available (< 5 min) to inform clinicians whether or not to commence ECPR for both OHCA and IHCA. However, such criteria also inherently reduce specificity, potentially resulting in the initiation of cardiac arrest patients who are unlikely to benefit. Future work on refining criteria for OHCA and developing specific criteria for IHCA is a high priority for future ECPR research.

## Conclusion

Adherence to different selection criteria impacts both the ECPR survival rate and the total number of survivors. Commonly used selection criteria may be unsuitable to select IHCA ECPR patients.

### Supplementary Information

**Additional file 1:** The following lists the definitions used for ‘end-stage disease’ or a similarly worded criterion in the original publications.

## Data Availability

The data supporting the conclusions of this article is included within the article and its additional file.

## Abbreviations

CO_2_: Carbon dioxide
CPC: Cerebral performance category
ECPR: Extracorporeal cardiopulmonary resuscitation
ELSO: Extracorporeal Life Support Organisation
IHCA: In-hospital cardiac arrest
OHCA: Out-of-hospital cardiac arrest
PaO2: Arterial oxygen tension
ROSC: Return of spontaneous circulation
