# Supplementary material for: The effect of restrictive versus liberal selection criteria on survival in ECPR: a retrospective analysis of a multi-regional dataset
Source: Scand J Trauma Resusc Emerg Med. 2023 Dec 4;31:89. doi: 10.1186/s13049-023-01154-1 (PMC10694924; doi:10.1186/s13049-023-01154-1)
Supplement: Supplementary file 1 — Additional file 1: The following lists the definitions used for ‘end-stage disease’ or a similarly worded criterion in the original publications. [file 13049_2023_1154_MOESM1_ESM.docx]

***Additional file***

*Article title*

**The effect of restrictive vs liberal selection criteria on survival in ECPR: A retrospective analysis of a multi-regional dataset.**

*Authors*

Arne Diehl, MD^1,2^

Andrew C. Read^3^

Timothy Southwood^4^

Hergen Buscher^3^

Mark Dennis^5,6^

Vinodh Bhagyalakshmi Nanjayya^1,2^

Aidan J. C. Burrell, PhD^1,2^

1 Department of Intensive Care and Hyperbaric Medicine, The Alfred Hospital, Melbourne, Australia

2 Australian and New Zealand Intensive Care Research Centre (ANZIC-RC), Dept. of Epidemiology and Preventive Medicine, Monash University, Melbourne, Australia

3 Department of Intensive Care, St Vincent’s Hospital, Sydney, Australia

4 Department of Intensive Care, Royal Prince Alfred Hospital, Sydney, Australia

5 Department of Cardiology, Royal Prince Alfred Hospital, Sydney, Australia

6 Faculty of Medicine and Health, University of Sydney, Sydney, Australia

***Correspondence***

Arne Diehl, MD FCICM FACEM

Department of Intensive Care and Hyperbaric Medicine, The Alfred, Commercial Road, Melbourne VIC 3004, Australia, a.diehl@alfred.org.au , phone +61 418342638, fax +613 9076 3676

**Additional Table A.1**

**Definition of end-stage disease**

The following lists the definitions used for ‘end-stage disease’ or a similarly worded criterion in the original publications.

PRAGUE trial

- Confirmed pregnancy
- Obvious life-limiting comorbidities
- Bleeding diathesis
- Known do-not resuscitate order
- Known prearrest Cerebral Performance Category (CPC)15 3 or greater

INCEPTION trial

- Terminal heart failure (NYHA III or IV)
- Severe pulmonary disease (COPD GIII of GIV)
- Disseminated oncological disease
- Obvious or suspected pregnancy
- Bilateral femoral bypass surgery
- Known contraindications for ECPR
- Known pre-arrest CPC-score of 3 or 4
- Known limitations in therapy or a Do Not Resuscitate-order

|  |  |  |  |  |  |
| --- | --- | --- | --- | --- | --- |

ARREST trial

- Valid do not resuscitate orders
- Nursing home resident
- Presence of an opt-out study bracelet
- Known pregnancy
- Terminal cancer

CHEER trial

- Known significant pre-existing neurological disability.
- Non-cardiac co-morbidities that cause limitations in activities of daily living e.g. severe chronic airways disease, cirrhosis of the liver, renal failure on dialysis and terminal illness due to malignancy.

The Alfred

- Severe disability impairing activities of daily living
- End-stage organ disease – cardiac, liver, lung, renal
- Other life-limiting diseases e.g. malignancy, terminal illness
- Advanced healthcare directive

**Additional Table A.2**

**Additional baseline patient characteristics**

| Characteristics | Total (n=199) | In-hospital (n=104) | Out of hospital (n=95) | P-value |
| --- | --- | --- | --- | --- |
| *Details of ECMO run* | | | | |
| Location of initiation  Emergency Department, *n (%)*  Intensive Care, *n (%)*  Cardiac catheterization laboratory, *n (%)*  Operating theatre, *n (%)*  Ward or other in-hospital location, *n (%)* | 113 (57.1)  55 (27.8)  21 (10.6)  7 (3.5)  3 (1.5) | 24 (23.3)  55 (53.4)  16 (15.5)  6 (5.8)  3 (2.9) | 89 (93.7)  0 (0)  5 (5.3)  1 (1.1)  0 (0) |  |
| *Cardiac arrest* | | | | |
| Witnessed by ambulance (out of hospital) , *n (%)* |  | 0 (0) | 14 (14.7) |  |
| Bystander CPR within 5 minutes*, n (%)* | 180 (90.5) | 102 (98.1) | 78 (82.1) | <0.001 |
| Signs of life reported,  *n* (%) | 34 (17.1) | 14 (13.5) | 20 (21.1) | 0.16 |
| EtCO2, n=66*, median (IQR)* | 30 (18-37) | 30 (19-35) | 30 (16-41) | 0.69 |
| Adrenaline dose in mg, n=190, *median (IQR)* | 5 (2-9) | 3 (2-4) | 8 (6-11) | <0.001 |
| Amiodarone dose in mg, n=190, *median (IQR)* | 0 (0-300) | 0 (0-150) | 300 (0-450) | <0.001 |
| Defibrillations, *median (IQR)* | 2 (0-5) | 0 (0-2) | 5 (2-9) | <0.001 |
| Intubated before start of ECMO cannulation, *n(%)* | 155 (77.9) | 77 (74) | 78 (82.1) | 0.17 |
| No known end-stage disease, *n (%)* | 173 (87.3) | 83 (79.8) | 90 (94.7) | 0.002 |
| New diagnosis of end-stage disease, *n (%)* | 8 (4) | 6 (5.8) | 2 (2.1) | 0.19 |
| *Retrospective cause of presumed cardiac arrest* | | | | |
| Cardiac cause total, *n (%)*  Acute myocardial infarction on angiogram,  *n* (%)  Vasospasm on angiogram,  *n* (%)  Primary rhythm disorder, *n* (%)  Cardiomyopathy, *n* (%)  Other cardiac causes, *n* (%) | 77 (38.9)  7 (3.5)  9 (4.6)  11 (5.6)  39 (19.7) | 33 (31.7)  4 (3.6)  4 (3.6)  6 (5.8)  27 (26) | 44 (46.8)  3 (3.2)  5 (5.3)  5 (5.3)  12 (12.8) |  |
| Pulmonary embolism,  *n* (%) | 15 (7.6) | 11 (10.6) | 4 (4.3) |  |
| Aortic pathology,  *n* (%) | 4 (2.0) | 1 (1.0) | 3 (3.2) |  |
| Sepsis,  *n* (%) | 6 (3.0) | 5 (4.8) | 1 (1.1) |  |
| Uncertain cause,  *n* (%) | 30 (15.2) | 13 (12.5) | 17 (18.1) |  |
| *Outcome* | | | | |
| CPC at 3 months, n=62,  *n* (%)  1  2  3  4  No follow up | 49 (79)  4 (6.5)  1 (1.6)  0 (0)  8 (12.9) | 35 (81.4)  2 (4.7)  0 (0)  0 (0)  6 (14) | 14 (73.7)  2 (10.5)  1 (5.3)  0 (0)  2 (10.5) |  |
| Cause of death – predominant cause, n=137  Multi-organ failure,  *n* (%)  Brain death,  *n* (%)  Poor neurological outcome – palliated,  *n* (%)  No cardiac recovery – palliated,  *n* (%)  Other,  *n* (%) | 40 (29.2)  20 (14.6)  21 (15.3)  21 (15.3)  35 (25.6) | 20 (32.8)  5 (8.2)  10 (16.4)  13 (21.3)  13 (21.3) | 20 (26.3)  15 (19.7)  11 (14.5)  8 (10.5)  22 (29) |  |

IQR, interquartile range; *cardiac arrest time refers to the longest arrest time with or without ROSC<20min; CPR, cardiopulmonary resuscitation; EtCO2, end-tidal carbon dioxide; CPC, cerebral performance category.

**Additional Table A.3**

Data table underlying figure 4

**OHCA**

|  | CHEER | The Alfred | ARREST | INCEPTION | Prague |
| --- | --- | --- | --- | --- | --- |
| sensitivity | 0.37 | 0.53 | 0.79 | 0.79 | 0.84 |
| specificity | 0.88 | 0.82 | 0.62 | 0.47 | 0.25 |

**IHCA**

|  | CHEER | The Alfred | ARREST | INCEPTION | Prague |
| --- | --- | --- | --- | --- | --- |
| sensitivity | 0.23 | 0.37 | 0.44 | 0.37 | 0.63 |
| specificity | 0.92 | 0.75 | 0.75 | 0.79 | 0.48 |
